# Usability of CHA_2_DS_2_VASC score in predicting the effectiveness and safety of pharmacological cardioversion – data from the multicenter cardioversion with intravenous ANTazoline study

**DOI:** 10.3389/fcvm.2025.1648549

**Published:** 2025-10-20

**Authors:** Bartosz Krzowski, Krzysztof Ozierański, Michał Peller, Wojciech Wróbel, Dawid Miśkowiec, Edyta Ćwiek-Rębowska, Aleksander Maciąg, Michał Farkowski, Marek Szołkiewicz, Beata Ceynowa-Sielawko, Marek Koziński, Maciej Wójcik, Robert Błaszczyk, Hanna Szwed, Jarosław Kasprzak, Katarzyna Mizia-Stec, Maciej Wybraniec, Paweł Balsam

**Affiliations:** ^1^ First Chair and Department of Cardiology, Medical University of Warsaw, Warsaw, Poland; ^2^ “Club 30” of the Polish Cardiac Society, Poland; ^3^ First Department of Cardiology, School of Medicine in Katowice, Medical University of Silesia, Upper Silesia Medical Center, Katowice, Poland; ^4^ Department of Cardiology, Medical University of Lodz, Łódź, Poland; ^5^ Second Department of Heart Arrhythmia, National Institute of Cardiology, Warsaw, Poland; ^6^ Department of Cardiology, Ministry of Interior and Administration National Medical Institute, Warsaw, Poland; ^7^ Department of Cardiology and Angiology, Kashubian Center for Heart and Vascular Diseases, Pomeranian Hospitals, Wejherowo, Poland; ^8^ Department of Cardiology, Kociewie Health Center, Starograd Gdański, Poland; ^9^ First Department of Cardiology, Medical University of Gdańsk, Gdańsk, Poland; ^10^ Department of Cardiology and Cardiac Surgery, Medical University of Lublin, Lublin, Poland; ^11^ Department of Coronary Artery Disease and Cardiac Rehabilitation, National Institute of Cardiology, Warsaw, Poland

**Keywords:** pharmacological cardioversion, atrial fibillation, antazoline, CHA_2_DS_2_VASc, emergency medecine

## Abstract

**Background:**

Pharmacological cardioversion (PCV) is one of the therapeutic options within rhythm control strategies for atrial fibrillation (AF). Data on clinical determinants influencing its effectiveness and safety in real-world settings remain limited.

**Methods:**

This study is a sub-analysis of the multicenter Cardioversion with Intravenous Antazoline in Atrial Fibrillation II (CANT II) registry. The registry retrospectively included consecutive patients with recent-onset AF undergoing urgent PCV in six Polish centers between 2019 and 2020. We analyzed 931 patients stratified according to CHA₂DS₂-VASc score: Group I (0–1 points; *n* = 194), Group II (2–4 points; *n* = 580), and Group III (≥5 points; *n* = 157). The primary endpoint was successful restoration of sinus rhythm within 12 h, and the secondary endpoint was a composite of adverse events (death, syncope, bradycardia, hypotension).

**Results:**

The median age was 69 years, 48% were men, and the median CHA₂DS₂-VASc score was 3. Antazoline was used in 59% of patients, amiodarone in 53%, and propafenone in 19%. Sinus rhythm was restored in 69%, 70%, and 64% of patients in Groups I, II, and III, respectively (*p* = 0.45). The overall adverse event rate was low (2.1%) and did not differ significantly between groups (*p* = 0.16). Antazoline was most effective in Group I, while propafenone showed higher efficacy in Group II.

**Conclusions:**

In this retrospective sub-analysis of the CANT II registry, success of pharmacological cardioversion of AF is not associated with number of comorbidities as assessed by the CHA2DS2VASc score. PCV remains a feasible and generally safe option in emergency and cardiology department practice.

## Introduction

Atrial fibrillation (AF) is the most common supraventricular arrhythmia worldwide, with a rising prevalence. Consequently, the number of patients presenting to the emergency department with AF episodes is expected to increase. It is estimated that the lifetime risk of developing AF is 1 in 3 (1). According to the recent ESC guidelines (1), (electrical or pharmacological) cardioversion should be considered in symptomatic patients as a part of rhythm control strategy. Pharmacological approach seems to be less effective acutely in comparison to electrical cardioversion (2), but has several other advantages including avoiding necessity of fasting, sedation, anesthesia, and shortened hospitalisation, which translates to financial benefits and reduced risk of hospital-acquired infections.

The success rate of pharmacological cardioversion (PCV), defined as the restoration of sinus rhythm without adverse events, varies among individuals. To date, ECG parameters have been associated with successful PCV (3). Virk et al. demonstrated that dyslipidemia and left ventricular ejection fraction (LVEF) < 40% were associated with failure to achieve cardioversion after the first dose of dofetilide in patients with AF (4).

Individual thrombo-embolic risk assessment using CHA_2_DS_2_-VASc scale until 2024 and CHA_2_DS_2_-VA scale later according to European guidelines is an integral part of AF patients care (1). CHA_2_DS_2_-VASc is still recommended according to American guidelines (5). The CHA₂DS₂-VASc score reflects not only the risk of thromboembolic events but also summarizes selected comorbidities. A pooled individual patient meta-analysis has established that the CHA₂DS₂-VASc score predicts early recurrence of AF within the first 30 days following electrical or PCV (6). The question whether it could be used as a predictor for successful PCV remains not addressed.

Thus, this sub-analysis of the CANT II study (Cardioversion with ANTazoline in Atrial Fibrillation II registry) aimed to assess the impact of the CHA₂DS₂-VASc score on the success rate of PCV and its correlation with PCV-related adverse events.

## Methods

### Study population

This study is a retrospective sub-analysis of the Cardioversion with Intravenous Antazoline in Atrial Fibrillation II (CANT II) registry. The registry collected consecutive patients with recent-onset atrial fibrillation (AF) undergoing urgent pharmacological cardioversion (PCV) across six centers in Poland between June 2019 and February 2020. For the present analysis, we included only patients with complete data on the CHA₂DS₂-VASc score and clinical outcomes. Of the 1,365 patients originally registered, 434 were excluded due to incomplete baseline or follow-up data, leaving 931 patients in the final study cohort.

Eligible patients were adults with paroxysmal or short-duration persistent AF, treated in the emergency department or cardiology ward. Patients were excluded if they had missing data necessary to calculate the CHA₂DS₂-VASc or CHA₂DS₂-VA score, incomplete documentation of pharmacological treatment, or unavailable information regarding cardioversion outcomes or safety endpoints. The study's rationale, design, and main results have been previously described in detail (7).

### Endpoints

The primary endpoint was successful restoration of sinus rhythm, confirmed by a 12-lead electrocardiogram. The secondary endpoint was a composite of safety outcomes, including bradycardia (<45 bpm), hypotension (systolic blood pressure drop >40 mmHg), syncope, or death.

### Stratification by risk scores

For the main analysis, patients were stratified into three groups according to their CHA₂DS₂-VASc score: Group I (0–1 points), Group II (2–4 points), and Group III (≥5 points). In an additional analysis, patients were also stratified using the CHA₂DS₂-VA score, which excludes sex from risk assessment, in line with the 2024 ESC guidelines.

The study protocol complied with the Declaration of Helsinki guidelines and was approved by the Ethics Committee of the Medical University of Silesia in Katowice (approval number KNW/022/KB1/9/18, issued on 13 February 2018). Informed consent was obtained from all subjects.

#### Statistical analysis

The distribution of continuous variables was assessed using the Shapiro–Wilk test. Non-normally distributed continuous variables are presented as medians with interquartile ranges, while categorical variables are expressed as percentages.

Statistical significance of trends across increasing CHA₂DS₂-VASc score groups was assessed using the Jonckheere–Terpstra test for quantitative variables and the Cochran-Armitage test for trend for qualitative variables. Fisher's exact test was applied for categorical variable comparisons, while the Mann–Whitney U test was used for continuous variable comparisons in independent groups. A *P*-value of <0.05 was considered statistically significant. Statistical analyses were conducted using SAS (Statistical Analysis Software, Cary, NC, USA), version 9.4.

## Results

### Patients' characteristics

A total of 931 patients with complete CHA₂DS₂-VASc data were included in the analysis. The median age of the study population was 69 [61–79] years, and 48% of participants were men. The median AF episode duration was 10 [4–24] hours, while the median CHA₂DS₂-VASc score was 3 [2–4] points. The majority of patients (62%) were in Group II. Patients in Group III were more likely to have AF episodes lasting >7 days (*p* = 0.014). However, they had shorter hospital stays (*p* < 0.001), higher body weight (*p* = 0.014), lower creatinine levels (*p* = 0.004), more comorbidities, and were less frequently hospitalized (*p* < 0.001). Patients in this group were also more likely to receive amiodarone (*p* = 0.027) and less likely to be treated with propafenone (*p* = 0.013). Overall, antazoline was administered to 59% of patients, amiodarone to 53%, and propafenone to 19%. The characteristics of the study population are presented in Table 1. Additional analysis has been performed according to CHA_2_DS_2_VA score. See detail in Supplementary Table S1.

**Table 1:** General population characteristics according to predefined groups (I - CHA2DS2VASc = 0–1; II - CHA2DS2VASc = 2–4; III - CHA2DS2VASc ≥4).

Characteristic	Overall study population	I - CHA2DS2VASc = 0-1	II - CHA2DS2VASc = 2-4	III - CHA2DS2VASc ≥4	*p*-value
Number of patients	931	194 (21%)	580 (62%)	157 (17%)	–
Age (years)	69 [61–76]	51 [46–61]	69 [64–75]	77 [71–80]	<0.001
Men	446/931 (48%)	165/194 (85%)	246/580 (42%)	35/157 (22%)	<0.001
AF episode duration (hours)	10 [4–24]	10 [4–24]	12 [5–25]	12 [6–24]	0.08
Time of admission (hour of the day)	13 [10–18]	13 [10–18]	13 [10–18]	9 [13–17]	0.62
Days of hospitalization	1 [1–2]	1 [1–1]	1 [1–2]	1 [1–3]	<0.001
BMI [kg/m^2^]	28.09 [25.33–31.71]	29.08 [25.37–31.65]	27.94 [25.09–32.28]	28.21 [25.52–31.02]	0.61
EHRA class	3 [2–3]	2 [2–3]	3 [2–3]	3 [2–3]	0.21
Heart rate (beats per minute)	114 [100–130]	120 [100–130]	120 [100–138]	110 [100–130]	0.84
Body weight (kg)	80 [70–90]	87.5 [73.5–97]	80 [69–90]	76.5 [70–85]	0.014
Height (cm)	167 [160–175]	174 [165–178]	167 [160–174]	162 [159–170]	<0.001
Age >65 years	557/931 (60.90%)	12/194 (6.19%)	406/580 (70.00%)	149/157 (94.90%)	<0.001
Age >75 years	237/931 (25.46%)	0/194 (0%)	138/580 (23.79%)	99/157 (63.06%)	<0.001
HT	702/927 (75.73%)	75/193 (38.86%)	473/577 (81.98%)	154/157 (98.09%)	<0.001
HR >130/min	273/878 (31.09%)	53/180 (29.44%)	176/546 (32.23%)	44/152 (5.01%)	0.972
PAD	274/929 (29.49%)	16/192 (8.33%)	161/580 (26.03%)	107/157 (68.15%)	<0.001
Stroke/TIA	64/929 (6.89%)	0/0 (0%)	21/580 (3.62%)	43/157 (27.39%)	<0.001
LaD (mm)	44 [40–47]	40 [38–46]	43 [41–47]	45 [41–48]	<0.001
Troponin (ng/ml)	0.011 [0.007–0.0195]	0.007 [0.005–0.012]	0.011 [0.007–0.0185]	0.016 [0.010–0.028]	<0.001
Creatinine (mg/dl)	0.99 [0.82–1.16]	0.95 [0.85–1.08]	0.97 [0.80–1.16]	1.06 [0.87–1.32]	0.004
eGFR (ml/min)	72.27 [56.0–86.0]	88.90 [75.0–90.0]	71.27 [57.0–83.0]	73.00 [43.97–73.0]	<0.001
Potassium (mEq/L)	4.24 [3.95–4.50]	4.30 [4.00–4.55]	4.20 [3.75–4.51]	4.30 [4.00–4.59]	0.71
WBC (k/mm^3^)	7.55 [6.30–9.04]	7.30 [6.21–8.68]	7.48 [6.21–8.81]	8.19 [6.65–9.73]	0.007
Hemoglobin (g/dl)	14.3 [13.1–15.3]	15.3 [14.6–16.2]	14.2 [13.1–15.1]	13.4 [12.2–14.4]	<0.001
TSH (uIU/ml)	1.86 [1.07–2.97]	1.91 [1.13–2.82]	1.82 [1.06–2.85]	1.89 [1.05–3.20]	0.94
History of AF ablation	69/926 (7.45%)	21/192 (11.98%)	41/579 (7.08%)	5/155 (3.23%)	0.002
AF episode lasting >7 days	103/896 (11.50%)	17/183 (9.29%)	58/559 (10.38%)	28/154 (18.18%)	0.014
Admission to the hospital	454/918 (49.46%)	76/189 (40.21%)	270/573 (47.12%)	108/156 (69.23%)	<0.001
Previous anticoagulant treatment	651/897 (72.58%)	106/186 (56.99%)	425/559 (76.03%)	120/152 (78.95%)	<0.001
VKA	174/792 (21.97%)	23/154 (14.94%)	116/493 (23.53%)	35/145 (24.14%)	0.051
DOAC	485/805 (60.25%)	86/160 (53.75%)	314/499 (62.93%)	85/146 (58.22%)	0.385
TEE	55/823 (6.28%)	9/166 (5.42%)	34/508 (6.69%)	12/149 (8.05%)	0.351
KIG	448/921 (48.64%)	103/193 (53.37%)	274/574 (61.16%)	71/154 (46.10%)	0.161
Beta-blocker	303/911 (33/26%)	63/189 (33.33%)	190/568 (33.455)	50/154 (32.47%)	0.876
Amiodarone	495/931 (53.17%)	89/194 (45.88%)	316/580 (54.48%)	90/157 (57.32%)	0.027
Propafenone	174/931 (18.69%)	50/194 (25.77%)	99/580 (17.07%)	25/157 (15.92%)	0.013
Phenazoline	551/931 (59.18%)	115/194 (59.28%)	334/580 (57.59%)	102/157 (64.97%)	0.333

AF, atrial fibrillation; BMI, body mass index; EHRA, European Heart Rhythm Association; DOAC, direct oral anticoagulant; eGFR, estimated glomerular filtration rate; HT, hypertension; HR, heart rate; KIG, potassium and glucose; LaD, left atrial diameter; LVEF, left ventricular ejection fraction; PAD, peripheral artery disease; TEE, transesophageal echocardiography; TIA, transient ischemic attack; TSH, thyroid stimulating hormone; WBC, white blood count; VKA, vitamin K antagonist.

### Endpoints

**Figure 1:**
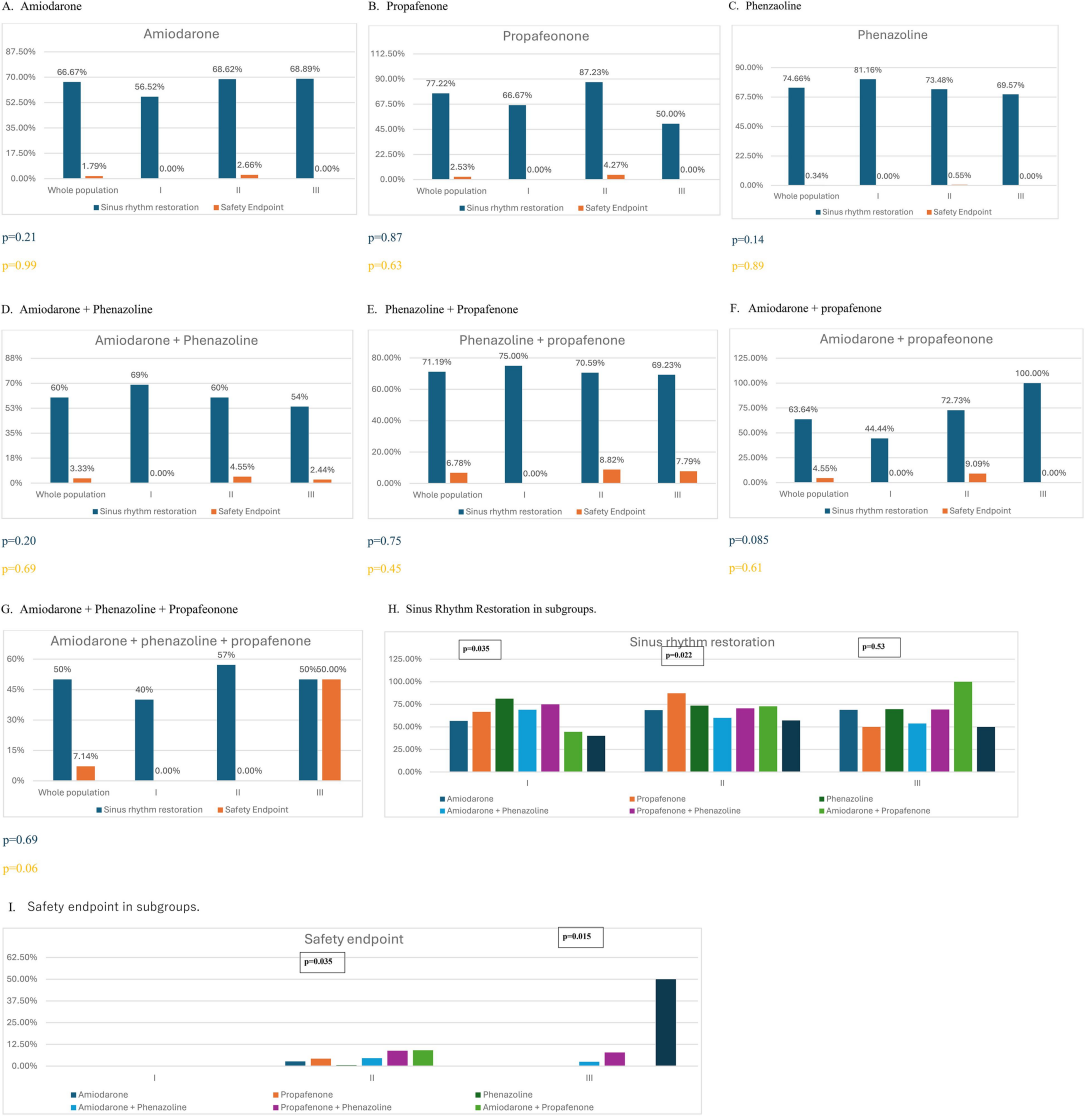
Effectiveness and safety of pharmacological cardioversion with different antiarrhythmic drugs according to predefined group (I - CHA2DS2VASc = 0–1; II - CHA2DS2VASc = 2–4; III - CHA2DS2VASc ≥4).

Sinus rhythm was restored with PCV in 68.85% of the overall study population and in 69%, 70%, and 64% of patients in Groups I, II, and III, respectively. There were no statistically significant differences in the sinus rhythm restoration rate between groups (*p* = 0.45). Events classified as safety endpoints occurred in 2.1% of the analyzed population, with no significant differences observed between groups (*p* = 0.16). Quantitative data on PCV efficacy and safety across all groups are presented in Table 2. Additional analysis has been performed according to CHA_2_DS_2_VA score. See detail in Supplementary Table S2. The most effective drug in Group I was antazoline (*p* = 0.035), while propafenone was the most effective in Group II (*p* = 0.022). In Group III, there were no statistically significant differences in sinus rhythm restoration effectiveness between drugs (*p* = 0.53). There were no safety endpoint events in Group I. In Group II, the administration of amiodarone + propafenone was associated with the highest rate of adverse events (*p* = 0.035). In Group III, adverse events most frequently occurred after administration of amiodarone + propafenone + antazoline (*p* = 0.015). See Figure 1 for details.

**Table 2:** Pharmacological cardioversion effectiveness and safety (I - CHA2DS2VASc = 0–1; II - CHA2DS2VASc = 2–4; III - CHA2DS2VASc ≥4).

Characteristic	Overall study population	I - CHA2DS2VASc = 0-1	II - CHA2DS2VASc = 2-4	III - CHA2DS2VASc ≥4	*p*-value
Sinus rhythm restoration	68.85% 641/931	68.56% 133/194	70.17% 407/580	64.33% 101/157	0.45
Safety endpoint	2.15% 20/931	0% 0/194	2.93% 17/580	1.91% 3/157	0.16

## Discussion

The prevalence of AF is increasing and will result in more patients presenting with acute episodes (1). A personalized approach is crucial to optimizing therapy for individual patients, ensuring better utilization of available healthcare resources.

AF is associated with increased morbidity, a higher risk of ischemic events, and an elevated likelihood of heart failure development. However, from the patients' perspective, AF episodes can significantly impair quality of life (1). The symptoms vary among individuals. Patients with palpitations often seek help in emergency departments, where those with severe conditions are also admitted for immediate care. Electrical cardioversion is more effective (∼90%) than PCV (∼70%), but PCV is often attempted first as it avoids anesthesia and may shorten hospital stay (8). Possible pitfalls of administering antiarrhythmic drugs include increased risk of proarrhythmia in patients with structural heart disease (Vaughan–Williams class Ic) (9), high cost and low availability (vernakalant) (10), or delayed onset of action in the case of amiodarone (11), which can cause longer stay in the Emergency Department and the need for a potentially preventable hospital admission.

Individual thromboembolic risk assessment is a cornerstone of care for patients with AF. The CHA₂DS₂-VASc score has been recommended for years to evaluate the need for oral anticoagulation therapy (12). CHA_2_DS_2_-VASc is still recommended according to American guidelines (5). Only recently authors of the 2024 ESC Guidelines recommended the use of updated scale CHA2DS2-VA (1). CHA₂DS₂-VASc remains central for thromboembolic risk assessment. Whether it could also predict PCV success had not been studied before. Vitali et al, conducted systemic review and individual patient pooled meta-analysis on the correlation between CHA2DS2-VASc score and risk of AF recurrence after successful cardioversion. Vitali et al. conducted a systematic review and an individual patient-pooled meta-analysis to examine the correlation between the CHA₂DS₂-VASc score and the risk of AF recurrence after successful cardioversion. Data from nearly 3,000 patients were analyzed. The CHA₂DS₂-VASc score was found to be a predictor of early AF recurrence within the first 30 days following either electrical or pharmacological cardioversion (6). However, no success rate or risk of complication in terms of CHA2DS2-VASc has been analyzed.

The CANT II Study appears to be well-suited for assessing the potential association between the CHA₂DS₂-VASc score and the likelihood of successful and safe PCV. Previous analyses from this registry have demonstrated the good efficacy of PCV with antazoline (7, 8, 13), but also explored sex-related differences in terms of pharmacological cardioversion (14) and safety of antazoline administration in patients with chronic kidney disease (15). Those projects were consequences of the previous studies on smaller populations showing good efficacy of the antazoline in patients with AF episode (16), which confirmed the widespread belief regarding antazoline utility in clinical practice.

Unfortunately, CHA2DS2-VASc score failed to correlate with the success rate of PCV and the rate of adverse events in our analysis. Overall success rate was close to 70%, which is similar to results reported previously (8). On the other hand, the rate of dangerous events related to drug administration was low and similar to values reported earlier (17), suggesting that this procedure is safe and might be worth giving a shot in certain circumstances. Vinoalas et al. established lack of obesity (body mass index < 30 kg/m^2^), duration of AF < 1 year and the absence of structural heart disease to be independent variables with predictive value of pharmacological reversal to sinus rhythm (18), which are not directly included into CHA_2_DS_2_-VASc score.

It is worth emphasizing that antazoline was the most effective drug in Group I, while propafenone was the most effective in Group II. Drug combinations appeared to be less effective, likely because they were administered following the failure of a single-agent treatment. On the other hand, complications were most frequently observed after the administration of AAD combinations in patients with higher CHA₂DS₂-VASc scores. These findings provide valuable insight into optimizing therapy selection, aligning with current guidelines for personalized AF management (1).

Other clinical parameters have also been investigated in relation to successful PCV. Zeemering et al. analyzed AF complexity using 12-lead ECGs, which significantly improved the prediction of both successful cardioversion and progression to persistent AF compared to conventional clinical and echocardiographic predictors (3). Dawood, on the other hand, highlighted the high variability in response to antiarrhythmic drugs, emphasizing the growing importance of pharmacogenetics in the management of AF (19). Moreover, the growing role of artificial intelligence in medicine, particularly in cardiology, could enhance the identification of factors associated with successful cardioversion in emergency departments. This advancement has the potential to assist both physicians and patients in optimizing AF treatment and improving clinical outcomes.

### Limitations

This analysis has several limitations, primarily due to its retrospective design. The exact number of screened patients who were excluded is unknown, as the study was conducted within a registry framework. However, the exclusion criteria were strictly applied to identify consecutive patients diagnosed with ICD-10 code I48 who underwent AF PCV, were not using antiarrhythmic agents, and met anticoagulation eligibility criteria for PCV. It should be acknowledged that mild adverse events related to drug administration may have been underreported. Additionally, a significant number of patients with structural heart disease received propafenone and antazoline, which is not aligned with current guidelines, potentially impacting the clarity of the conclusions. Finally, the study did not include other AADs, such as vernakalant, ibutilide, or flecainide, as these medications were unavailable in Poland at the time of data collection.

## Conclusions

The risk of adverse events associated with PCV or its effectiveness does not appear to correlate with the CHA₂DS₂-VASc score. PCV itself demonstrates high success rates and a favorable safety profile in patients with AF. It is important to individualize the treatment according to individual comorbidities. Further studies are needed to identify the determinants of successful and safe PCV. The results of this analysis may serve as a foundation for meta-analyses, increasing statistical power and enabling the identification of key risk factors for the described clinical endpoints.

## Data Availability

The raw data supporting the conclusions of this article will be made available by the authors, without undue reservation.
