# Supplementary material for: Usability of CHA2DS2VASC score in predicting the effectiveness and safety of pharmacological cardioversion – data from the multicenter cardioversion with intravenous ANTazoline study
Source: Front Cardiovasc Med. 2025 Oct 20;12:1648549. doi: 10.3389/fcvm.2025.1648549 (PMC12580191; doi:10.3389/fcvm.2025.1648549)

Figure S1. Effectiveness and safety of pharmacological cardioversion with different antiarrhythmic drugs according to predefined group (I - CHA2DS2VA = 0-1; II - CHA2DS2VA = 2-4; III - CHA2DS2VA ≥4).

A. Amiodarone

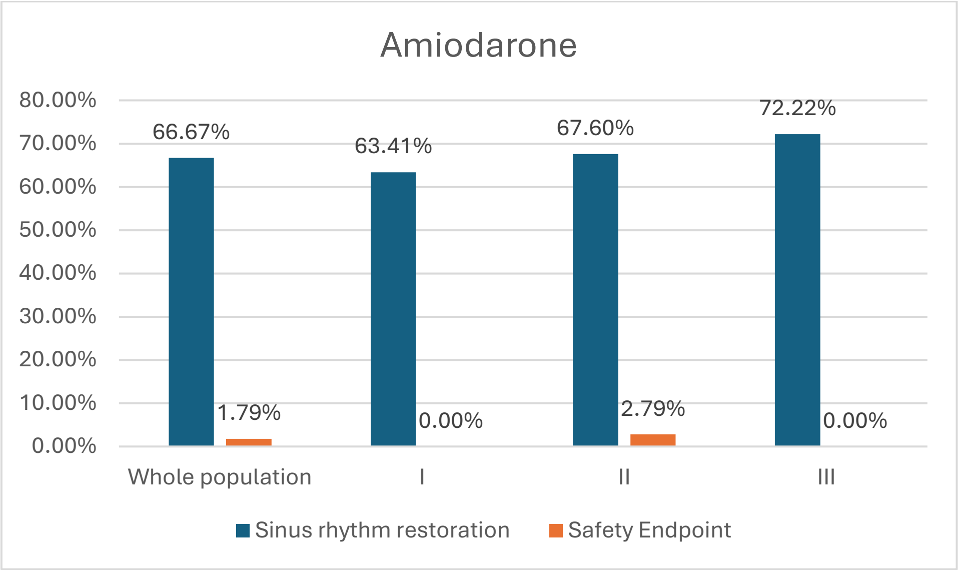

p=0.39

p=0.35

B. Propafenone

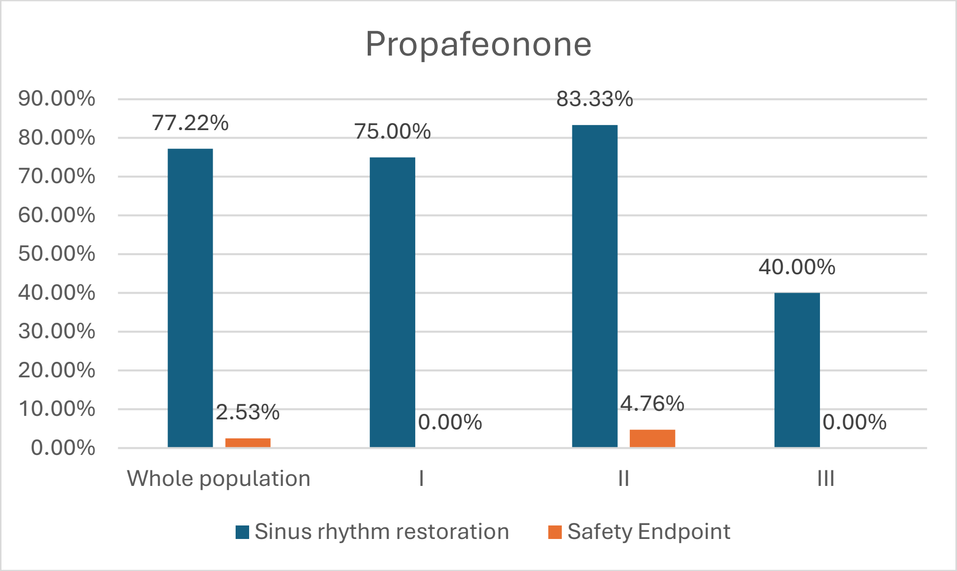

p=0.6

p=0.41

C. Phenzaoline

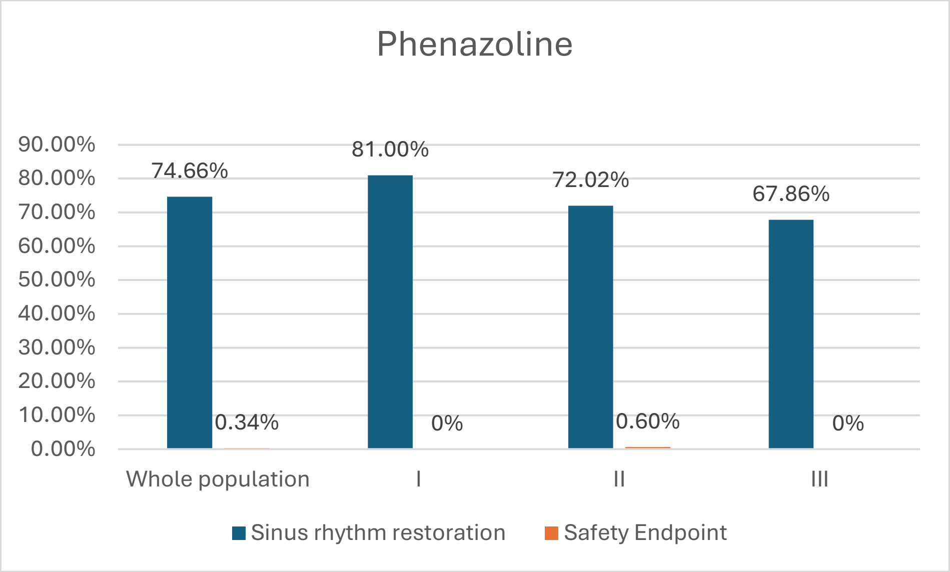

p=0.07

p=0.69

D. Amiodarone + Phenazoline

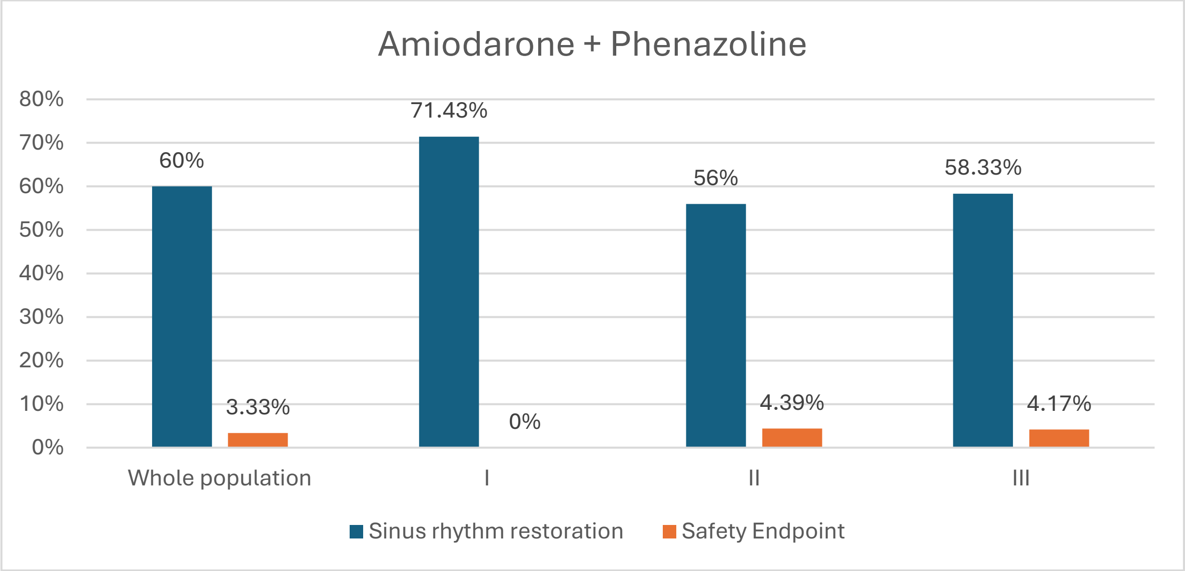

p=0.19

p=0.27

E. Phenazoline + Propafenone

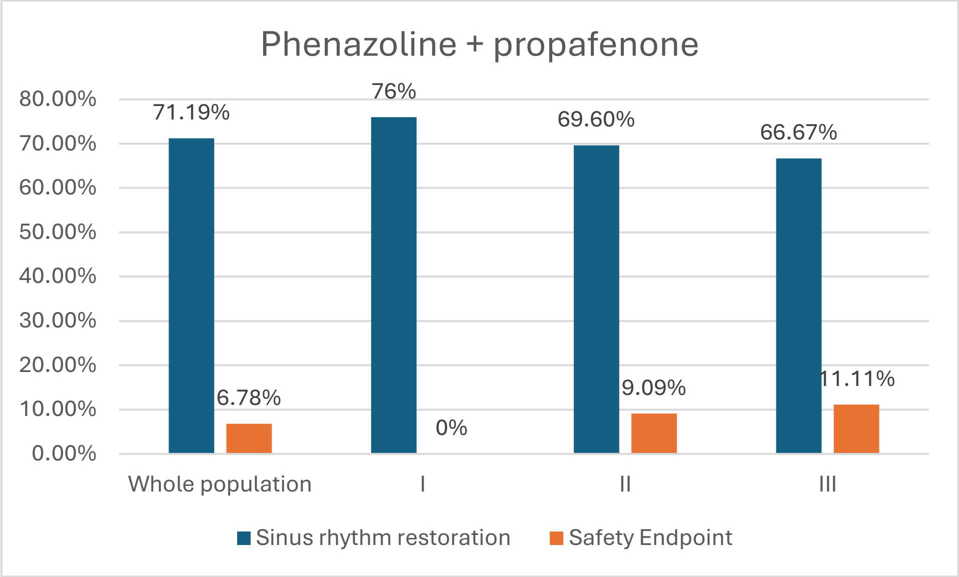

p=0.56

p=0.22

F. Amiodarone + propafenone

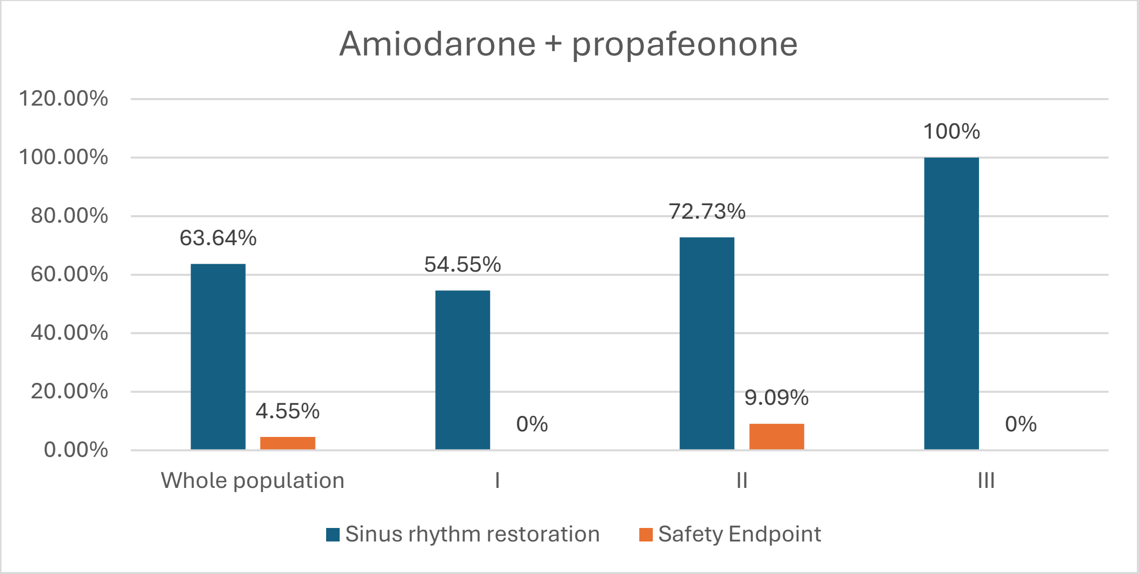

p=0.37

p=0.31

G. Amiodarone + Phenazoline + Propafeonone

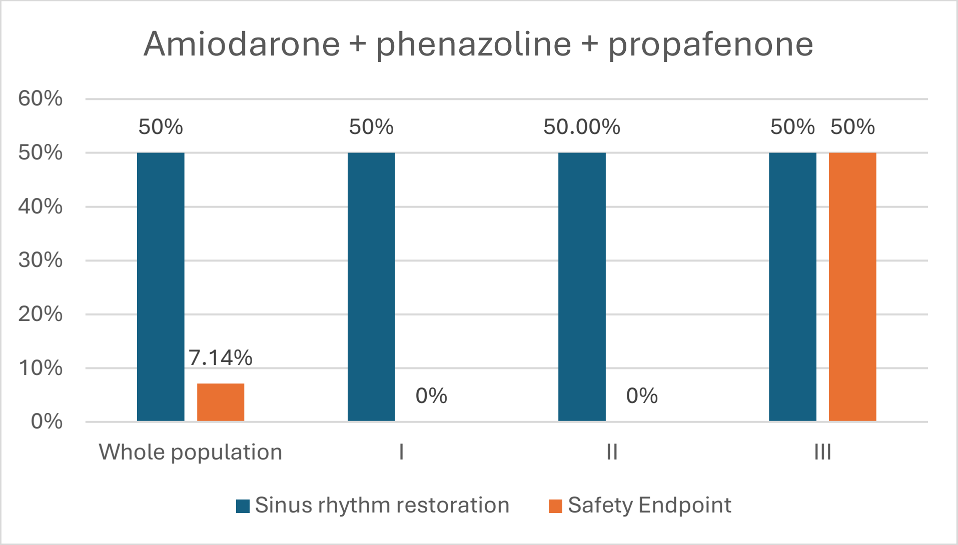

p=1.0

p=0.11

H. Sinus Rhythm Restoration in subgroups.

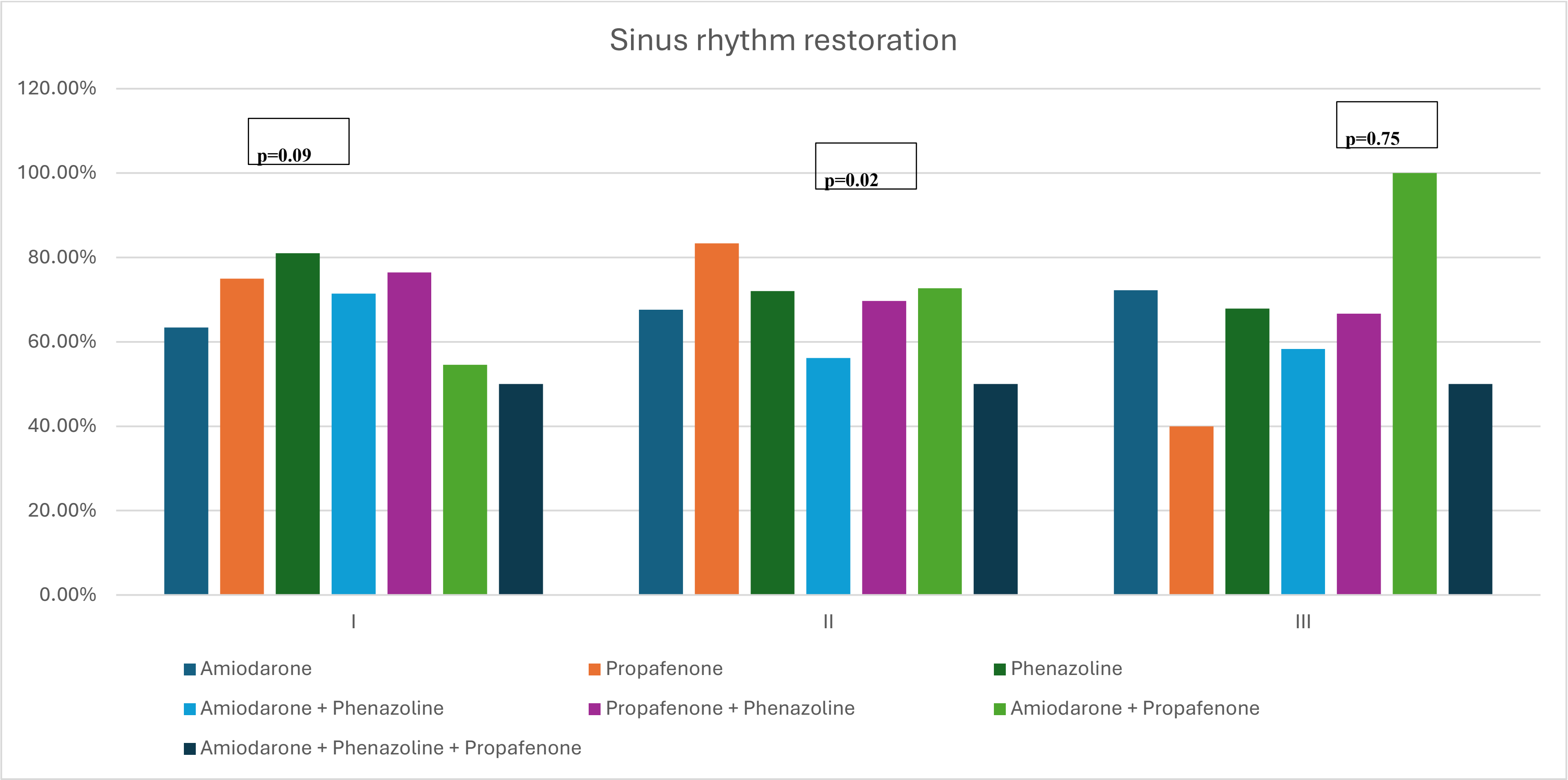

I. Safety endpoint in subgroups.

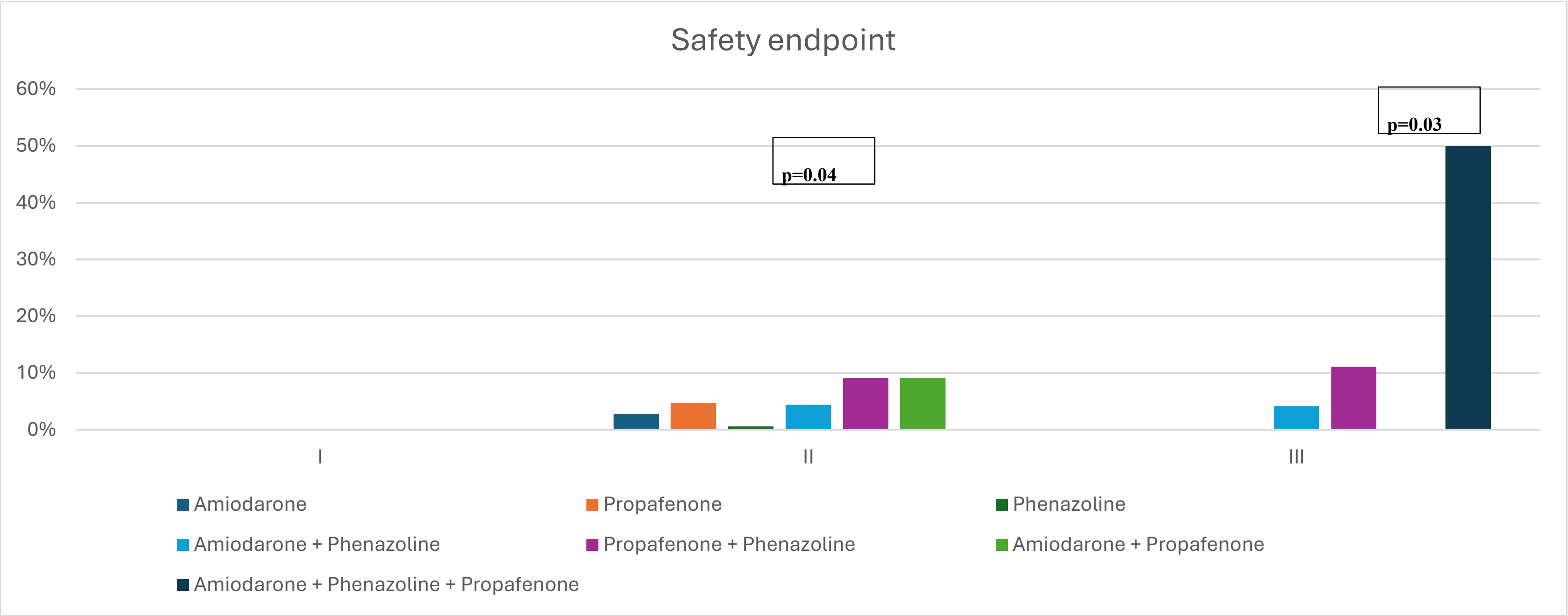

Supplement: Supplementary file 1 [file Image1.pdf]
