# Supplementary material for: Usability of CHA2DS2VASC score in predicting the effectiveness and safety of pharmacological cardioversion – data from the multicenter cardioversion with intravenous ANTazoline study
Source: Front Cardiovasc Med. 2025 Oct 20;12:1648549. doi: 10.3389/fcvm.2025.1648549 (PMC12580191; doi:10.3389/fcvm.2025.1648549)
Supplement: Supplementary file 2 [file Datasheet1.docx]

Figure legends

Figure S1. Effectiveness and safety of pharmacological cardioversion with different antiarrhythmic drugs according to predefined group (I - CHA2DS2VA = 0-1; II - CHA2DS2VA = 2-4; III - CHA2DS2VA ≥4).

Table S1. General population characteristics according to predefined groups (I - CHA2DS2VA = 0-1; II - CHA2DS2VA = 2-4; III - CHA2DS2VA ≥4).

|  | Overall study population | I | II | III | p-value |
| --- | --- | --- | --- | --- | --- |
| Number of patients | 931 | 290  (31.15%) | 555  (59.61%) | 86  (9.23%) | - |
| Age (years) | 69 [61-76] | 57 [50-64] | 71 [66-77] | 77.5 [74-81] | <0.001 |
| Men | 446/931 (48%) | 173/290  (59.66%) | 239/555  (43.06%) | 34/86  (39.53%) | <0.001 |
| AF episode duration (hours) | 1. [4-24] | 10 [5-24] | 12 [5-26] | 12 [6-24] | 0.79 |
| Time of admission (hour of the day) | 13 [10-18] | 13 [10-18] | 13 [10-18] | 12 [9-17] | 0.06 |
| Days of hospitalization | 1 [1-2] | 1 [1-1] | 1 [1-2] | 1 [1-4] | <0.001 |
| BMI [kg/m^2^] | 28.09 [25.33-31.71] | 27. 63 [24.72 – 31.56] | 29.16 [25.63 – 32.43] | 27.17 [24.87 – 29.41] | 0.57 |
| EHRA class | 3 [2-3] | 3 [2-3] | 3 [2-3] | 3 [2-3] | 0.31 |
| Heart rate (beats per minute) | 114 [100-130] | 120 [100-140] | 120 [100-130] | 110 [100-125] | 0.06 |
| Body weight (kg) | 80 [70-90] | 81 [70-95] | 80 [70-90] | 75 [69-80] | 0.11 |
| Height (cm) | 167 [160-175] | 170 [160-178] | 166 [158-173] | 165 [160-172] | 0.08 |
| Age >65 years | 567/931 (60.90%) | 53/290  (18.29%) | 431/555  (77.66%) | 83/86  (96.51%) | <0.001 |
| Age >75 years | 237/931 (25.46%) | 1/290  (0.34%) | 176/555  (74.26%) | 60/86  (69.77%) | <0.001 |
| HT | 702/927 (75.73%) | 130/288  (45.14%) | 487/553  (88.07%) | 85/86  (98.84%) | <0.001 |
| HR >130/min | 273/878 (31.09%) | 91/273  (33.33%) | 166/522  (31.8%) | 16/83  (19.28%) | 0.052 |
| PAD | 274/929 (29.49%) | 20/288  (6.94%) | 179/555  (32.25%) | 75/86  (87.21%) | <0.001 |
| Stroke/TIA | 64/929 (6.89%) | 0/288  (0%) | 30/555 (5.41%) | 34/86  (39.53%) | <0.001 |
| LaD (mm) | 44 [40-47] | 42 [38-45] | 44 [41-47] | 46 [43-49] | <0.001 |
| Troponin (ng/mL) | 0.011 [0.007-0.0195] | 0.008 [0.005-0.012] | 0.012 [0.007 – 0.019] | 0.02 [0.014 – 0.033] | <0.001 |
| Creatinine (mg/dL) | 0.99 [0.82-1.16] | 0.93 [0.81-1.08] | 0.99 [0.82 – 1.18] | 1.11 [0.91 – 1.35] | <0.001 |
| eGFR (mL/min) | 72.27 [56.0-86.0] | 84 [69 – 90] | 68.31 [54 – 82] | 55.5 [43.97 – 73.92] | <0.001 |
| Potassium (mEq/L) | 4.24 [3.95-4.50] | 4.3 [3.9-4.54] | 4.28 [4 – 4.51] | 4.3 [3.98 – 4.55] | 0.82 |
| WBC (k/mm^3^) | 7.55 [6.30-9.04] | 7.3 [6.1 – 8.69] | 7.56 [6.3 – 8.99] | 8.34 [6.7 – 9.73] | 0.007 |
| Hemoglobin (g/dL) | 14.3 [13.1-15.3] | 15 [14 – 16] | 14.2 [13 – 15.1] | 13.4 [12.2 – 14.4] | <0.001 |
| TSH (uIU/mL) | 1.86 [1.07-2.97] | 1.9 [1.16 – 2.77] | 1.84 [1.04 – 2.96] | 1.19 [1.22 – 3.18] | 0.98 |
| History of AF ablation | 69/926 (7.45%) | 31/88  (10.76%) | 36/553  (6.51%) | 2/85  (2.9%) | 0.004 |
| AF episode lasting >7 days | 103/896 (11.50%) | 22/274  (8.03%) | 68/537 (12.66%) | 13/85  (15.29%) | 0.026 |
| Admission to the hospital | 454/918 (49.46%) | 108/284  (38.03%) | 282/548  (51.46%) | 64/86  (74.42%) | <0.001 |
| Previous anticoagulant treatment | 651/897 (72.58%) | 168/275  (61.09%) | 419/540  (77.59%) | 64/82  (78.05%) | <0.001 |
| VKA | 174/792 (21.97%) | 39/229  (17.03%) | 116/485  (23.92%) | 19/78  (24.36%) | 0.056 |
| DOAC | 485/805 (60.25%) | 133/237  (56.12%) | 307/489  (62.78%) | 45/79  (56.96%) | 0.38 |
| TEE | 55/823 (6.28%) | 12/243  (4.94%) | 33/498  (6.63%) | 10/82  (12.2%) | 0.041 |
| KIG | 448/921 (48.64%) | 158/288  (54.86%) | 252/547  (46.07%) | 38/86  (44.19%) | 0.016 |
| Beta-blocker | 303/911 (33/26%) | 97/282  (34.4%) | 177/543  (32.6%) | 29/86  (33.72%) | 0.74 |
| Amiodarone | 495/931 (53.17%) | 141/290  (48.62%) | 310/555  (55.85%) | 44/86  (51.16%) | 0.21 |
| Propafenone | 174/931 (18.69%) | 66/290  (22.76%) | 92/555  (16.58%) | 16/86  (18.6%) | 0.09 |
| Phenazoline | 551/931 (59.18%) | 165/290  (56.9%) | 323/555  (58.2%) | 63/86  (73.26%) | 0.036 |

AF – atrial fibrillation; BMI – body mass index; EHRA - European Heart Rhythm Association; DOAC – direct oral anticoagulant; eGFR – estimated glomerular filtration rate; HT – hypertension; HR – heart rate; KIG – potassium and glucose; LaD – left atrial diameter; LVEF – left ventricular ejection fraction; PAD – peripheral artery disease; TEE – transesophageal echocardiography; TIA – transient ischemic attack; TSH – thyroid stimulating hormone; WBC – white blood count; VKA - vitamin K antagonist;

Table S2. Pharmacological cardioversion effectiveness and safety.

|  | Whole population | I | II | III | p-value |
| --- | --- | --- | --- | --- | --- |
| Sinus rhythm restoration | 68.85% 641/931 | 72.06%  209/290 | 67.93%  377/555 | 63.95%  55/86 | 0.11 |
| Safety endpoint | 2.15% 20/931 | 0%  0/290 | 3.06%  17/555 | 3.49%  3/86 | 0.005 |
